# Cesarean delivery on maternal request and common child health outcomes: A prospective cohort study in China

**DOI:** 10.7189/jogh.12.11001

**Published:** 2022-02-26

**Authors:** Ke-yi Si, Hong-tian Li, Yu-bo Zhou, Zhi-wen Li, Le Zhang, Ya-li Zhang, Rong-wei Ye, Jian-meng Liu

**Affiliations:** 1 Institute of Reproductive and Child Health, National Health Commission Key Laboratory of Reproductive Health, Peking University Health Science Center, Beijing, China; 2 Department of Epidemiology and Biostatistics, School of Public Health, Peking University Health Science Center, Beijing, China; 3 Department of Health Statistics, Naval Medical University, Shanghai, China

## Abstract

**Background:**

Cesarean delivery vs vaginal delivery was reported to increase the risks of childhood obesity, pneumonia, anemia, and neurobehavioral disorders, but few studies were able to deal with the confounding biases associated with medical conditions indicating cesareans. This prospective cohort study aims to investigate the associations of non-medically indicated cesarean delivery on maternal request (CDMR) with these child health outcomes.

**Methods:**

Among 17 748 liveborn infants whose mothers (primiparas) participated in a randomized controlled trial on micronutrient supplementation and pregnancy outcomes during 2006-2009 in 5 rural counties in Hebei Province, China, 6972 singletons born by full-term spontaneous vaginal delivery (SVD) and 3626 by CDMR were extracted for the assessments of obesity (weight-for-height z-score >3) and pneumonia (self-reported) at 1.5-5 years in 2011. Some children were further randomly selected from these two groups for the assessments of anemia (hemoglobin <110 g/L, 2341 SVD and 2417 CDMR) and neurobehavioral disorders (raw score of Child Behavior Checklist larger than the 90th percentile of the normative sample, 1257 SVD and 1060 CDMR).

**Results:**

Compared with SVD, CDMR was associated with increased risks of obesity (adjusted odds ratio (aOR) = 1.41, 95% confidence interval (CI) = 1.14-1.75, *P* = 0.002) and anemia (aOR = 1.65, 95% CI = 1.28-2.12, *P* < 0.001), but not with the risk of pneumonia (aOR = 1.16, 95% CI = 0.94-1.45, *P* = 0.17) or neurobehavioral disorders (aORs varied from 0.82 to 0.91, *P* > 0.05) in childhood.

**Conclusions:**

Cesarean delivery, independent of cesarean indications, is likely associated with childhood obesity and anemia, indicating a need to keep pregnant women informed, especially those seeking CDMR, a need to explore possible improvement on obstetric service, and even a need for main stakeholders to reach a compromise in making a cesarean decision.

**Trial registration:**

ClinicalTrials.gov: NCT00133744 and NCT01404416

Cesarean section (CS) is a life-saving surgery when medically indicated. CS rate has increased dramatically from 12.1% in 2000 to 21.1% in 2015 globally and from 28.8% in 2008 to 36.7% in 2018 in China, much higher than the threshold (15%) recommended by the World Health Organization, implying an unnecessary use of CS [1,2]. This phenomenon has raised great concern especially when accumulated evidence indicated potential adverse impacts of CS on child health. Several meta-analyses of observational studies reported that cesarean-delivered children had higher risks of developing asthma, obesity, and type 1 diabetes than their vaginal counterparts [3,4]. In addition, some individual observational studies have linked CS with pneumonia, anemia, and neurobehavioral disorders in childhood [5-7]. However, it was difficult to distinguish whether the association reflected intrinsic impacts of CS or was confounded by related indications, because the medical indications (eg, macrosomia) that lead to CS may also lead to poor child health [8]. Comparisons between cesarean delivery on maternal request (CDMR) and vaginal delivery (VD) might be optimal for addressing this problem.

CDMR was defined as a full-term singleton cesarean delivery (CD) in the absence of any medical indications [9]. Women request CS mainly owing to fear of labor pain, fear of poor-quality care during VD, fear of bad quality of life after VD, perceived safety of CS, self-determined timing of birth, etc. [10]. Maternal overweight/obesity is another reason for CDMR [11]. Meanwhile, the health professionals and hospitals may prefer to agree with women’s request due to fear of medical litigation, financial incentives, inadequate skills for performing an assisted VD, etc. [10]. What’s more, in China, although the total cost of CS is higher than that of VD, the reimbursements offered by the government narrow the cost gap, which may further lower the barriers to request a CS [12]. CDMRs were estimated to account for 0.9% to 56% of all CDs across different countries, among which China had the highest proportion [13]. The increased use of CDMR over time was proposed as an important driver of the total increase in primary cesarean rate [14].

Compared with overall/elective CD mixed with various indications, the traditional subjects used to investigate the association of CS with child health, the health status of pregnant women and their fetuses in CDMR is more comparable to that in VD, both unaffected by indications, such as serious pregnancy complications and macrosomia. Thus, the impacts of CDMR on child health can be attributed to CS per se and/or factors accompanying CS. However, few studies have been able to identify CDMR in the current medical coding classification systems. To fill this gap, we conducted a large-scale prospective cohort study in China to investigate the associations of CDMR with multiple adverse child health outcomes that were previously suggested, including obesity, pneumonia, anemia, and some neurobehavioral disorders. The study is highly merited in a public health perspective, not only because these childhood conditions are common and their impacts on health are long-lasting, but also because CDMR is very likely avoidable [13,15].

## METHODS

### Data and population

This prospective cohort study was conducted based on a randomized controlled trial implemented in 5 rural counties in Hebei Province, China. In the original trial, a total of 18 775 primiparas with hemoglobin >100 g/L prior to their 20th gestational week were recruited from May 2006 to April 2009, and randomly assigned to supplement folic acid, iron-folic acid, or multiple micronutrients from early pregnancy to delivery. Study details have been described elsewhere [16]. In 2011, among all live-born infants (N = 17 748) in the trial, 8258 singletons born by full-term spontaneous VD (SVD) and 4195 by CDMR were extracted and followed up for a physical examination at 1.5-5 years. Limited by the resource, 5000 children who still lived in the original counties were randomly selected from these two groups of children for the assessments of anemia, and 2700 for the Child Behavior Checklist (CBCL) test (Text S1 in the **Online Supplementary Document**). Both the original trial and the follow-up study were approved by the Peking University Health Science Center Institutional Review Board with identifiers of NCT00133744 and NCT01404416 on Clinicaltrials.gov, respectively. All participants provided written informed consent.

### Exposures, outcomes, and covariates

Delivery mode, the exposure of interest, was defined based on information about delivery mode, cesarean indications, gestational age, and membrane status in the medical records. The original delivery modes were categorized as SVD, assisted VD (assisted breech, breech extraction, vacuum extraction, or forceps), elective CD (before the onset of labor), emergency CD (after the onset of labor), or others. For CD, indications were delineated as maternal complications, cephalopelvic disproportion, macrosomia, breech presentation, transverse lie, fetal distress, placenta previa, maternal request, or other factors. To implement the current study, SVD and CDMR (full-term elective CD indicated by maternal request without premature rupture of membranes) were selected from the available population [9]. To enhance the comparability of SVD with CDMR, participants in the SVD group were also restricted to full-term births.

The primary outcome of this study was obesity at 1.5-5.0 years, and the secondary outcomes included pneumonia, anemia, and internalizing (emotional)/externalizing (behavioral)/total problems at 1.5-5.0 years. Child’s height and weight were measured by trained physicians at township health centers using a tailored height board with precision to the nearest 0.1 cm and an electronic scale (BW-150, UWE, Beijing, China) to the nearest 50 g, respectively. Height boards and scales were periodically calibrated. Obesity was defined as weight-for-height greater than 3 standard deviations above the World Health Organization Child Growth Standards median [17]. Pneumonia was confirmed if the caregiver reported that the child had been diagnosed with pneumonia by hospitals of township level or above. Children’s hemoglobin was tested using a photometric instrument (Model 201; HemoCue). Anemia was defined as hemoglobin <110 g/L. Besides, the CBCL was filled out by the caregiver to assess the neurobehavioral development. The clinical range for internalizing/externalizing/total problems in the CBCL referred to raw score larger than the 90th percentile of the normative sample [18]. Detailed interpretation of the score is shown in Text S2 in the **Online Supplementary Document**.

Most of covariates were extracted from the database of the original trial, including supplements received during pregnancy, maternal age at delivery, education, occupation, gestational age, body mass index (BMI) in the 1st trimester, gestational weight gain (GWG) rate in the 2nd/3rd trimester [19], hemoglobin in mid-pregnancy, level of delivery hospital, child’s gender, and birth weight. Child’s age at the follow-up visit, feeding pattern before 6 months old, and medical insurance status were derived from the follow-up questionnaire.

### Statistical analysis

All analyses were performed using R software (version 4.0.3). The *P* values were considered significant at <0.05 (two-sided). Continuous variables are presented as means and standard deviations or as medians and interquartile ranges, and categorical variables as frequencies and percentages. Differences between the CDMR and the SVD groups were examined using Student’s *t*-test or Mann-Whitney U test for continuous variables and χ^2^ test for categorical variables. To study the association of CDMR with outcomes of interest, odds ratios (ORs) and 95% confidence intervals (CIs) were estimated using multivariable logistic regression. For all outcomes, models were adjusted for maternal age at delivery (year, continuous), education (≤primary, secondary, or ≥ high school), occupation (farmer or not), gestational age (week, continuous), BMI in the 1st trimester (<18.5, 18.5-22.9, 23.0-27.4, or ≥27.5 kg/m^2^, cut-offs for Asians) [20], GWG rate (kg/week, in quintiles), micronutrient supplementation during pregnancy (folic acid, iron-folic acid, or multiple micronutrients), level of delivery hospital (provincial/city, county/district, or township/village level), and medical insurance status (yes or no); child’s gender (male or female), birth weight (g, continuous), age at the follow-up visit (month, continuous), and feeding pattern before 6 months old (exclusive breastfeeding, mixed feeding, or formula feeding). Besides, maternal anemia in mid-pregnancy (yes or no) was additionally adjusted for childhood anemia and neurobehavioral disorders. The proportion of missing information on these covariates was <5%. Missing data on GWG rate were first imputed with median and then made into categories. Participants with missing information on maternal anemia, level of delivery hospital, feeding pattern, and medical insurance status were imputed with the dominant category.

To examine the robustness of the analyses, we further conducted two sets of sensitivity analyses. First, a complete-case analysis was performed using pairwise deletion on missing covariates. Second, for obesity and pneumonia, comparisons of intent were made between full-term planned VD and planned CDMR with covariates imputed, as recommended by the 2006 National Institutes of Health Consensus panel [9]. The original intention for post-labor CD indicated by fetal distress or other factors was presumable VD. Therefore, the planned VD group consisted of SVD, assisted VD, and this subgroup of post-labor CD at term. Planned CDMR included full-term pre-/post-labor CD indicated by maternal request (Figure S1 in the **Online Supplementary Document**).

## RESULTS

**Figure 1:**
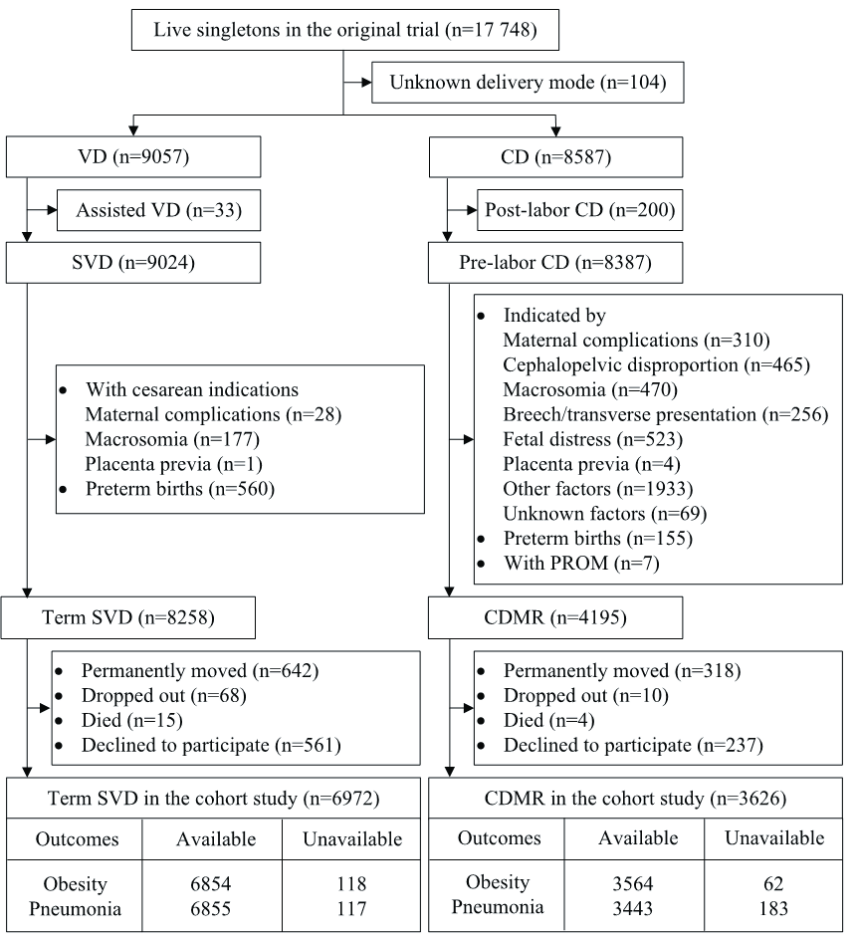
Flowchart of inclusion and exclusion. VD – vaginal delivery, SVD – spontaneous vaginal delivery, CD – cesarean delivery, CDMR – cesarean delivery on maternal request, PROM – premature rupture of membranes.

As outlined in **Figure 1**, of the 17 748 live singletons in the original trial, 8258 children delivered by SVD at term and 4195 children delivered by CDMR were identified as potential participants of the current cohort study. Among them, 960 (7.7%) permanently moved, 78 (0.6%) dropped out, and 19 (0.2%) died prior to the start of this study. Of the remaining, 798 (6.4%) declined to participate. After excluding those without information on outcomes, 10 418 (83.7%) and 10 298 (82.7%) children were finally included in the analyses for obesity and pneumonia. Similarly, 95.2% (4758/5000) and 85.8% (2317/2700) children remained in the analyses for anemia and CBCL, respectively, as outlined in Text S1 in the **Online Supplementary Document**. Maternal and offspring characteristics of the included and the excluded are presented in Table S1 in the **Online Supplementary Document****.** The characteristics by delivery mode are shown in **Table 1** and Table S2 in the **Online Supplementary Document**. Compared with women undergoing SVD, those undergoing CDMR were more likely to be overweight/obese in the 1st trimester and deliver in township/village hospitals.

**Table 1:** Maternal and offspring characteristics by mode of delivery in obesity analysis*

Characteristics	SVD (N = 6854)	CDMR (N = 3564)	*P-*value†
**Maternal details**			
Age at delivery, year, median (IQR)	22.8 (3.0)	22.9 (3.2)	0.008
Gestational age, week, mean (SD)	39.9 (1.2)	39.8 (1.2)	<0.001
Education, No. (%)			<0.001
≤Primary	1069 (15.6)	664 (18.6)	
Secondary	5694 (83.1)	2833 (79.5)	
≥High school	91 (1.3)	67 (1.9)	
Occupation, No. (%)			0.05
Farmer	6326 (92.3)	3249 (91.2)	
Others	528 (7.7)	315 (8.8)	
BMI in the 1^st^ trimester, kg/m^2^, No. (%)			<0.001
<18.5	683 (10.0)	253 (7.1)	
18.5-22.9	4615 (67.3)	2109 (59.2)	
23.0-27.4	1408 (20.5)	982 (27.6)	
≥27.5	148 (2.2)	220 (6.2)	
GWG rate in the 2^nd^/3^rd^ trimester, kg/week, mean (SD)	0.43 (0.17)	0.46 (0.18)	<0.001
Missing, No. (%)	225 (3.3)	81 (2.3)	
Supplementation during pregnancy, No. (%)			0.90
Folic acid	2269 (33.1)	1193 (33.5)	
Iron-folic acid	2317 (33.8)	1197 (33.6)	
Multiple micronutrients	2268 (33.1)	1174 (32.9)	
Level of delivery hospital, No. (%)			<0.001
Provincial/city	538 (7.8)	212 (5.9)	
County/district	5253 (76.6)	2588 (72.6)	
Township/village	1063 (15.5)	763 (21.4)	
Missing	0 (0)	1 (0.03)	
Medical insurance, No. (%)			0.01
Yes	6074 (88.6)	3215 (90.2)	
No	767 (11.2)	342 (9.6)	
Missing	13 (0.2)	7 (0.2)	
**Offspring details**			
Male	3543 (51.7)	1845 (51.8)	>0.999
Birth weight, g, No. (%)			0.001
<2500	84 (1.2)	19 (0.5)	
2500-3999	6770 (98.8)	3545 (99.5)	
Apgar score at 1 min, No. (%)			<0.001
0-7	93 (1.4)	9 (0.3)	
8-10	6758 (98.6)	3555 (99.7)	
Missing	3 (0.04)	0 (0)	
Feeding pattern before 6 months, No. (%)			0.90
Exclusive feeding	5659 (82.6)	2957 (83.0)	
Mixed feeding	966 (14.1)	497 (13.9)	
Formula feeding	216 (3.2)	107 (3.0)	
Missing	13 (0.2)	3 (0.1)	
Age at the follow-up visit, month, No. (%)			<0.001
18-29	921 (13.4)	735 (20.6)	
30-35	1550 (22.6)	929 (26.1)	
36-41	1287 (18.8)	695 (19.5)	
42-47	1856 (27.1)	746 (20.9)	
48-60	1240 (18.1)	459 (12.9)	

SVD – spontaneous vaginal delivery, CDMR – cesarean delivery on maternal request, BMI – body mass index, GWG – gestational weight gain, IQR – interquartile range, SD – standard deviation

*Percentages may not add up to 100% due to rounding.

**†**Comparisons between groups were made by Student’s *t*-test for gestational age and GWG rate, Mann-Whitney U test for maternal age, and χ^2^ test for other variables.

### Primary outcome

In this cohort, 6854 children were born by SVD and 3564 by CDMR, among which 397 (3.8%) were obese. Children born by CDMR were more likely to be obese than those born by SVD (4.5% vs 3.5%, crude OR = 1.31, 95% CI = 1.07-1.61, *P* = 0.009, **Table 2**). After multivariable adjustment, the OR was slightly increased to 1.41 (95% CI = 1.14-1.75, *P* = 0.002).

**Table 2:** Crude and adjusted odds ratios for multiple child health outcomes by mode of delivery

Outcomes	Delivery Mode	No. of Events/No. of Children (%)	Crude OR (95% CI)	*P*-value	Adjusted OR (95% CI)*	*P*-value	Adjusted OR (95% CI)†	*P*-value
Obesity	SVD	237/6854 (3.5)	1 [Reference]		1 [Reference]		1 [Reference]	
	CDMR	160/3564 (4.5)	1.31 (1.07-1.61)	0.009	1.36 (1.10-1.69)	0.005	1.41 (1.14-1.75)	0.002
Pneumonia	SVD	267/6855 (3.9)	1 [Reference]		1 [Reference]		1 [Reference]	
	CDMR	144/3443 (4.2)	1.08 (0.88-1.32)	0.48	1.14 (0.92-1.41)	0.24	1.16 (0.94-1.45)	0.17
Anemia	SVD	120/2341 (5.1)	1 [Reference]		1 [Reference]		1 [Reference]	
	CDMR	183/2417 (7.6)	1.52 (1.20-1.92)	<0.001	1.60 (1.25-2.06)‡	<0.001	1.65 (1.28-2.12)‡	<0.001
Internalizing Problems	SVD	133/1257 (10.6)	1 [Reference]		1 [Reference]		1 [Reference]	
	CDMR	91/1060 (8.6)	0.79 (0.60-1.05)	0.11	0.81 (0.60-1.08)‡	0.15	0.82 (0.61-1.10)‡	0.18
Externalizing Problems	SVD	53/1257 (4.2)	1 [Reference]		1 [Reference]		1 [Reference]	
	CDMR	37/1060 (3.5)	0.82 (0.54-1.26)	0.37	0.88 (0.56-1.38)‡	0.59	0.90 (0.57-1.41)‡	0.63
Total problems	SVD	97/1257 (7.7)	1 [Reference]		1 [Reference]		1 [Reference]	
	CDMR	72/1060 (6.8)	0.87 (0.64-1.20)	0.39	0.91 (0.65-1.26)‡	0.56	0.91 (0.65-1.26)‡	0.56

SVD – spontaneous vaginal delivery, CDMR – cesarean delivery on maternal request, OR – odds ratio, CI – confidence interval

*Adjusted for maternal age at delivery (year, continuous), education (≤primary, secondary, or ≥ high school), occupation (farmer or not), gestational age (week, continuous), body mass index in the 1st trimester (<18.5, 18.5-22.9, 23.0-27.4, or ≥27.5 kg/m^2^), gestational weight gain rate in the 2nd/3rd trimester (kg/week, in quintiles), and micronutrient supplementation (folic acid, iron-folic acid, or multiple micronutrients); child’s gender (male or female), birth weight (g, continuous), age at the follow-up visit (month, continuous), and feeding pattern before 6 mo old (exclusive breastfeeding, mixed feeding, or formula feeding).

**†**Additionally adjusted for level of delivery hospital (provincial/city, county/district, or township/village level) and medical insurance status (yes or no).

‡Additionally adjusted for maternal anemia in mid-pregnancy (yes or no).

### Secondary outcomes

The overall prevalence was 4.0% for pneumonia, 6.4% for anemia, 9.7% for internalizing problems, 3.9% for externalizing .problems, and 4.7% for total problems. Compared with SVD, CDMR was associated with a higher risk of anemia in childhood (7.6% vs 5.1%, adjusted OR = 1.65, 95% CI = 1.28-2.12, *P* < 0.001). There was no significant difference when comparing the risk of pneumonia (4.2% vs 3.9%, adjusted OR = 1.16, 95% CI = 0.94-1.45, *P* = 0.17), internalizing problems (8.6% vs 10.6%, adjusted OR = 0.82, 95% CI = 0.61-1.10, *P* = 0.18), externalizing problems (3.5% vs 4.2%, adjusted OR = 0.90, 95% CI = 0.57-1.41, *P* = 0.63), and total problems (6.8% vs 7.7%, adjusted OR = 0.91, 95% CI = 0.65-1.26, *P* = 0.56) between CDMR and SVD.

Results of the complete-case analysis and the intention-to-treat analysis were similar to those of the main analysis with covariates imputed (Table S3 and Table S4 in the **Online Supplementary Document**).

## DISCUSSION

In this prospective cohort study, non-medically indicated CDMR was associated with increased risks of obesity and anemia, yet did not affect the risk of pneumonia or neurobehavioral disorders in childhood, indicating that CS might be a risk factor for childhood obesity and anemia independent of cesarean indications.

### CDMR and childhood obesity

Many observational studies have investigated the association of CS with the risk of childhood obesity, but few of them were able to deal with the potential biases associated with cesarean indications [3,21]. In this study, we found that CDMR, without any medical indication, labor, or premature rupture of membranes, was associated with a 41% increased risk of obesity in children aged 1.5-5 years compared with SVD, suggesting that CS per se or its accompanying factors might increase the susceptibility for childhood obesity. This is consistent with the result of a previous cohort study, which indicated a borderline significant association (adjusted OR = 1.18, 95% CI = 1.00-1.41) between CDMR and overweight in Chinese children aged 3-7 years [22]. Another longitudinal study restricted to women without known risk factors for CS (maternal overweight/obesity, gestational diabetes, hypertensive disorders, etc.) also reported a significant association (adjusted relative risk = 1.30; 95% CI = 1.09-1.54) between CS and obesity in offspring followed from age 9-14 to age 20-28 years in the United States [23].

One of the biological mechanisms might be the distinct obesity-related intestinal microbiota profiles resulting from the circumvention of birth canal or the lack of labor, such as the decreased or delayed colonization of *Bifidobacteria*, *Bacteroides*, and *Collinsella*, and the increased quantities of *Firmicutes* [24-27]. The other potential mechanism revealed by a mouse experiment was that vasopressin, an appetite inhibitor, was less produced in cesarean-delivered offspring than the vaginally-born [28].

### CDMR and childhood pneumonia

In this study, children born by CDMR were at a non-significantly increased risk (16%) of pneumonia compared with those born by SVD. This may be due to our insufficient sample size (N = 10 298), because more than 35 500 participants are needed to detect an OR of 1.16 with 80% power when the incidence of pneumonia in the reference group is assumed to be 3.9%. Other studies with sufficient sample size all demonstrated that CD especially elective CD was associated with an elevated risk of childhood respiratory infection (8%-35%) [5,29-31].

The underlying mechanisms can be summarized as follows: cortisol, a cytokine promoting lung liquid clearance and lung maturation, was less generated in children born by elective CD [32]; the DNA methylation patterns related to immune responses in infants born by CD were different from those born vaginally [33]; the upper respiratory tract microbial profiles in children born by CD were less stable and abundant [34].

### CDMR and childhood anemia

A significant increased risk of anemia (65%) was observed in children aged 1.5-5.0 years born by CDMR. This supported the positive association between CDMR and anemia at 40-79 months (adjusted OR = 1.18) demonstrated by another Chinese cohort derived from a quite different socioeconomic setting [6]. A previous cohort study found that the iron-related hematological indices (serum ferritin, hemoglobin, red blood cell, and hematocrit) in cord blood were lower in CDMR than in SVD [35]. It is worth mentioning that no association was found between CDMR and anemia at 6 or 12 months in our previous study using the same population [6]. The inconsistency across age might be explained by the following reasons. The exclusion of women with hemoglobin ≤100 g/L at enrollment and the micronutrient supplementation throughout pregnancy might reduce the risk of anemia in early infancy, and narrow the gap of anemia between CDMR and SVD. As was seen in our previous study, the overall anemia prevalence at 6 and 12 months in this cohort was 6.8% and 5.3%, far below that of their peers in another study (26.4% at 6-11 months and 35.3% at 12-17 months) [36]. This gap might expand as children grew when the iron store/hemoglobin bonus endowed from these factors gradually faded out, which was verified by the post-hoc analysis, suggesting that the probability of anemia decreased as child’s age increased, but the rate of decline was slower for CDMR than for SVD (*P* < 0.001, Figure S2 in the **Online Supplementary Document**).

The observed higher risk of anemia in CDMR might be attributed to their less placental transfusion at birth because of early cord clamping, lack of utero squeezing and delayed onset of respiration [37]. A study found that the residual blood volume in umbilical cord after clamping was higher in CDMR than in SVD [35]. Notably, when this study was conducted, both groups underwent early cord clamping (<1 minute after birth), but the difference of placental transfusion may be enlarged after the universal implementation of delayed cord clamping (>1 minute after birth), because it is much easier to be followed in VD than in CD [38]. In addition, the gut microbiota has been linked to folate and vitamin B_12_ metabolism in humans, and iron sensing of intestinal cells in mice, all of which are associated with anemia [39-41]. Whether different profiles of gut microbiota between CD and VD lead to different risks of childhood anemia demands further study.

### CDMR and childhood neurobehavioral disorders

No significant associations were found between CDMR and neurobehavioral disorders recognized by CBCL (adjusted ORs varied from 0.82 to 0.91). Results from previous studies were inconsistent: no association (adjusted relative risk = 0.78; 95% CI = 0.47-1.37) was observed between CS and mental delay in preterm 2-year-olds in a cohort using Bayley Scales of Infant Development [42]; two studies using CBCL indicated that children born by CD had higher scores (worse performance) on internalizing problems than those born vaginally [43,44], whereas another study observed an opposite result [45]. Unlike diagnoses of the other outcomes, scores of these scales were served as a reference for preliminary screening. The validity of the results is largely dependent on the applicability of the scales to the population, which might lead to the high heterogeneity across studies.

A potential link between delivery mode and offspring neurobehavioral disorders is cortisol, a hormone closely related to cognitive functioning, which was less produced in cesarean-delivered children than those born vaginally when aged 6 months [46]. There were also some intriguing findings in animal experiments. For example, CS has been revealed to underlie alterations in the structure and function of the prefrontal cortex mediated by mitochondrial adaptations, manifesting as behavioral characteristics of psychiatric illness in mice [47]. Additionally, gene expression and cell death in rat/mouse brain varied significantly by delivery mode, but whether these can cause neurobehavioral disorders is undetermined [28].

### Strengths and limitations

This study has some strengths. Delicate classification of delivery mode and cesarean indications allowed us to identify CDMR and examine its intrinsic effect on child health. Children were restricted to full-term births and macrosomia were removed from the analyses, making the two delivery modes more comparable to each other. All participants were primiparas, eliminating potential confounding caused by birth order or parity-related complications [48]. Data on multiple critical covariates facilitated confounding adjustment. All information except for breastfeeding and medical insurance status were collected prospectively and the process of data collection and measurements was standardized, which minimized information bias.

This study also has several limitations. We did not have information about antepartum use of antibiotics, neonatal hormone levels, intestinal microbiota, or placental transfusion, therefore we were unable to explore whether they mediated the association of CDMR with child health. Additionally, we could not rule out the possibility of residual confounding induced by CDMR-specific characteristics. For example, as shown in **Table 1**, compared with women undergoing SVD, those undergoing CDMR had a greater BMI in the 1st trimester and a more substantial GWG rate in the 2nd/3rd trimester, and previous studies have reported that both maternal obesity during pregnancy and excessive GWG rate were risk factors for offspring obesity and anemia [19,49,50]. To reduce the potential impacts of residual confounding in relation to these two factors, we did a post-hoc analysis by restricting the obesity and anemia analyses to pregnant women who had normal BMI in the 1st trimester and normal GWG rate (the 2nd-4th quintiles) in the 2nd/3rd trimester and adjusted for continuous BMI and GWG rate in the model. Results showed that CDMR was associated with a significantly higher risk of childhood obesity after multivariable adjustment (adjusted OR = 1.43, 95% CI = 1.01-2.02, *P* = 0.04). Although the association was no longer significant for anemia due to the shrinking sample size (N = 1821), the point estimate was still larger than one (adjusted OR = 1.32, 95% CI = 0.79-2.20, *P* = 0.30), indicating the robustness of our analyses (Table S5 in the **Online Supplementary Document**). Another potential confounder, socioeconomic status, was unavailable in this study, but its proxies, such as education, occupation, level of delivery hospital, and medical insurance, were adjusted. However, characteristics that have been previously attributed to CDMR, such as antenatal maternal anxiety and mood disorders, were not assessed in this study, thus whether these may bias the association of interest is unknown [51]. Besides, we did not have objective indicators for the diagnosis of pneumonia, but the criteria were simple and consistent across different hospitals. Finally, women in this study were relatively well-nourished (hemoglobin >100 g/L at enrollment and took micronutrient supplements during pregnancy) and had good access to health care, which might limit the generalizability.

### Clinical and research implications

Findings in our study suggested that CS might increase the risks of childhood obesity and anemia independent of cesarean indications. It may be better to consider these impacts when deciding the delivery mode so that some unnecessary CS might be avoided. Meanwhile, some preventive strategies could be developed to mitigate the adverse impacts of CS. For example, CDMR was not recommended prior to 39 weeks of gestation to ensure lung maturity [9]; delayed cord clamping and umbilical cord milking were assessed to reduce the risk of offspring anemia [52]; vaginal seeding and probiotics supplementation were investigated to cope with microbiota dysbiosis caused by CD [53,54]; physical squeeze and administration of corticosteroids before elective CD were tried to imitate physiological environment induced by labor [55]. But these new interventions are in a fledging period, which merit further evaluations.

## CONCLUSION

CS, independent of cesarean indications, is likely associated with childhood obesity and anemia, indicating a strong need for informed decisions on delivery mode, especially for those seeking CDMR. The degree, duration, mechanisms, and countermeasures of the adverse effects of CS on child health demand further investigation.

## Additional material


Online Supplementary Document
