# Supplementary material for: Cesarean delivery on maternal request and common child health outcomes: A prospective cohort study in China
Source: J Glob Health. 2022 Feb 26;12:11001. doi: 10.7189/jogh.12.11001 (PMC8878985; doi:10.7189/jogh.12.11001)
Supplement: Online Supplementary Document [file jogh-12-11001-s001.pdf]

Online Supplementary Document

Text S1 Sampling strategy

Text S2 Brief introduction of the Child Behavior Checklist

Table S1 Comparisons of maternal and offspring characteristics between the included and the excluded

Table S2 Maternal and offspring characteristics by mode of delivery (for outcomes other than obesity)

Table S3 Crude and adjusted odds ratios for multiple child health outcomes by mode of delivery (complete case analysis)

Table S4 Crude and adjusted odds ratios for multiple child health outcomes by mode of delivery (intention-to-treat analysis)

Table S5 Crude and adjusted odds ratios for childhood obesity and anemia by mode of delivery (restricted to women who had normal BMI in the 1st trimester and normal gestational weight gain rate in the $2^{nd}/3^{rd}$ trimester)

Figure S1 Flowchart of inclusion and exclusion in the intention-to-treat analysis

Figure S2 Probability of anemia in different delivery modes by child's age

Text S1 Sampling strategy

Anemia

Among children who were alive and still lived in the 5 counties (Yuanshi, Fengrun, Mancheng, Laoting and Xianghe), 2500 children born by spontaneous vaginal delivery (SVD) and 2500 by cesarean delivery on maternal request (CDMR) were randomly selected from the original trial for the screening of anemia. After excluding children who refused to be tested, 2341 children born by full-term SVD and 2417 by CDMR were finally included in the analysis.

Neurobehavioral development

Among children who were alive and still lived in the 5 counties, 1200 children aged 18-30 months and 1500 children aged 31-60 months were randomly selected from the original trial for the Child Behavior Checklist (CBCL) test. Stratified sampling was carried out according to 5 counties and 2 kinds of delivery mode (SVD and CDMR). Ideally, 240 children aged 18-30 months and 300 aged 31-60 months would be randomly selected from each county. Since the number of children aged 18-30 months in Mancheng was the smallest among all the counties, children were selected according to the constituent ratio of two delivery modes in Mancheng from each county. For counties that did not have adequate eligible children, alternatives with similar age (±3 months) were randomly selected from the county that had the most children to make up the difference. After excluding children who declined to participate, whose Apgar score was less than 8, whose CBCL result was incomplete, 1257 children born by full-term SVD and 1060 by CDMR were included in the analysis for CBCL.

Text S2 Brief introduction of the Child Behavior Checklist

CBCL is a scale used to assess the emotional and behavioral problems in children aged 18-60 months. Caregivers are asked to rate 99 specific problem items and 1 open-ended problem item as 0 for not true of the children, 1 for somewhat or sometimes true, and 2 for very true or often true based on the children's behaviors in the last 2 months. These items are scored on 8 empirically based syndromes (emotionally reactive, anxious/depressed, somatic complaints, withdrawn, attention problems, aggressive behavior, sleep problems, and other problems). The first 4 syndromes are merged into an internalizing problem scale, the 5th and 6th as an externalizing problem scale, and all of the syndromes as a total problem scale. The total score in each scale is the raw score. Each raw score corresponds to a percentile of the normative sample. The clinical range of internalizing/externalizing/total problems is above the 90th percentile, the borderline clinical range is between the 83rd and 90th percentile, and the rest is in normal range.

Table S1 Comparisons of maternal and offspring characteristics between the included and the excluded*

Characteristics	Obesity		Pneumonia		Anemia		CBCL	
	**Excluded (n=2035)**	**Included (n=10 418)**	**Excluded (n=2155)**	**Included (n=10 298)**	**Excluded (n=242)**	**Included (n=4758)**	**Excluded (n=383)**	**Included (n=2317)**
**Maternal Details**								
Age at delivery, year, median (IQR)	23.3 (3.7)	22.8 (3.0)	23.3 (3.7)	22.8 (3.0)	22.8 (2.9)	22.9 (3.1)	22.9 (3.1)	22.7 (3.0)
Gestational age, week, mean (SD)	40.0 (1.1)	39.9 (1.2)	39.9 (1.1)	39.9 (1.2)	39.8 (1.2)	39.8 (1.2)	39.8 (1.5)	39.9 (1.2)
Education, No. (%)								
≤Primary	537 (26.4)	1733 (16.6)	554 (25.7)	1716 (16.7)	41 (16.9)	844 (17.7)	81 (21.1)	367 (15.8)
Secondary	1466 (72.0)	8527 (81.8)	1567 (72.7)	8426 (81.8)	194 (80.2)	3831 (80.5)	295 (77.0)	1923 (83.0)
≥High school	32 (1.6)	158 (1.5)	34 (1.6)	156 (1.5)	7 (2.9)	83 (1.7)	7 (1.8)	27 (1.2)
Occupation, No. (%)								
Farmer	1781 (87.5)	9575 (91.9)	1887 (87.6)	9469 (91.9)	216 (89.3)	4423 (93.0)	338 (88.3)	2169 (93.6)
Others	254 (12.5)	843 (8.1)	268 (12.4)	829 (8.1)	26 (10.7)	335 (7.0)	45 (11.7)	148 (6.4)
BMI in the 1st trimester, kg/m^2, No. (%)								
<18.5	187 (9.2)	936 (9.0)	203 (9.4)	920 (8.9)	18 (7.4)	408 (8.6)	42 (11.0)	193 (8.3)
18.5-22.9	1377 (67.7)	6724 (64.5)	1434 (66.5)	6667 (64.7)	158 (65.3)	2964 (62.3)	232 (60.6)	1453 (62.7)
23.0-27.4	411 (20.2)	2390 (22.9)	456 (21.2)	2345 (22.8)	55 (22.7)	1187 (24.9)	95 (24.8)	571 (24.6)
≥27.5	60 (2.9)	368 (3.5)	62 (2.9)	366 (3.6)	11 (4.5)	199 (4.2)	14 (3.7)	100 (4.3)
GWG rate in the 2nd/3rd trimester, kg/week, mean (SD)	0.41 (0.19)	0.44 (0.18)	0.41 (0.19)	0.44 (0.17)	0.45 (0.17)	0.44 (0.18)	0.45 (0.19)	0.43 (0.18)
Missing, No. (%)	71 (3.5)	306 (2.9)	71 (3.3)	306 (3.0)	10 (4.1)	133 (2.8)	18 (4.7)	82 (3.5)
Anemia in mid-pregnancy, No. (%)								
Yes	100 (4.9)	626 (6.0)	112 (5.2)	614 (6.0)	15 (6.2)	331 (7.0)	18 (4.7)	136 (5.9)
No	1922 (94.4)	9740 (93.5)	2031 (94.2)	9631 (93.5)	225 (93.0)	4401 (92.5)	361 (94.3)	2170 (93.7)
Missing	13 (0.6)	52 (0.5)	12 (0.6)	53 (0.5)	2 (0.8)	26 (0.5)	4 (1.0)	11 (0.5)

Table S1 Comparisons of maternal and offspring characteristics between the included and the excluded (continued)*

Characteristics	Obesity		Pneumonia		Anemia		CBCL	
	**Excluded (n=2035)**	**Included (n=10 418)**	**Excluded (n=2155)**	**Included (n=10 298)**	**Excluded (n=242)**	**Included (n=4758)**	**Excluded (n=383)**	**Included (n=2317)**
Supplementation during pregnancy, No. (%)								
Folic acid	675 (33.2)	3462 (33.2)	722 (33.5)	3415 (33.2)	79 (32.6)	1588 (33.4)	129 (33.7)	762 (32.9)
Iron-folic acid	668 (32.8)	3514 (33.7)	699 (32.4)	3483 (33.8)	80 (33.1)	1604 (33.7)	129 (33.7)	793 (34.2)
Multiple micronutrients	692 (34.0)	3442 (33.0)	734 (34.1)	3400 (33.0)	83 (34.3)	1566 (32.9)	125 (32.6)	762 (32.9)
Level of delivery hospital, No. (%)								
Provincial/city	143 (7.0)	750 (7.2)	146 (6.8)	747 (7.3)	21 (8.7)	245 (5.1)	22 (5.7)	175 (7.6)
County/district	1604 (78.8)	7841 (75.3)	1658 (76.9)	7787 (75.6)	196 (81.0)	3372 (70.9)	295 (77.0)	1720 (74.2)
Township/village	287 (14.1)	1826 (17.5)	350 (16.2)	1763 (17.1)	25 (10.3)	1140 (24.0)	66 (17.2)	421 (18.2)
Missing	1 (0.05)	1 (0.01)	1 (0.05)	1 (0.01)	0 (0)	1 (0.02)	0 (0)	1 (0.04)
**Offspring Details**								
Gender, No. (%)								
Male	1044 (51.3)	5388 (51.7)	1106 (51.3)	5326 (51.7)	126 (52.1)	2419 (50.8)	190 (49.6)	1178 (50.8)
Female	990 (48.6)	5030 (48.3)	1048 (48.6)	4972 (48.3)	116 (47.9)	2339 (49.2)	193 (50.4)	1139 (49.2)
Missing	1 (0.05)	0 (0)	1 (0.05)	0 (0)	0 (0)	0 (0)	0 (0)	0 (0)
Birth weight, g, No. (%)								
<2500	13 (0.6)	103 (1.0)	15 (0.7)	101 (1.0)	0 (0)	54 (1.1)	3 (0.8)	25 (1.1)
2500-3999	2022 (99.4)	10 315 (99.0)	2140 (99.3)	10 197 (99.0)	242 (100)	4704 (98.9)	380 (99.2)	2292 (98.9)

CBCL – Child Behavior Checklist, BMI – body mass index, GWG – gestational weight gain, IQR – interquartile range, SD – standard deviation

*Percentages may not add up to 100% due to rounding.

Table S2 Maternal and offspring characteristics by mode of delivery (for outcomes other than obesity)

Characteristics	Pneumonia			Anemia			CBCL		
	**SVD (n=6855)**	**CDMR (n=3443)**	***P* value**	**SVD (n=2341)**	**CDMR (n=2417)**	***P* value**	**SVD (n=1257)**	**CDMR (n=1060)**	***P* value**
**Maternal Details**									
Age at delivery, year, median (IQR)	22.8 (2.9)	22.9 (3.2)	0.01	22.8 (3.1)	23.0 (3.0)	0.01	22.6 (2.8)	22.9 (3.3)	0.01
Gestational age, week, mean (SD)	39.9 (1.2)	39.8 (1.2)	<0.001	39.9 (1.3)	39.7 (1.2)	<0.001	39.9 (1.3)	39.8 (1.2)	<0.001
Education, No. (%)			<0.001			<0.001			0.10
≤Primary	1073 (15.7)	643 (18.7)		365 (15.6)	479 (19.8)		188 (15.0)	179 (16.9)	
Secondary	5691 (83.0)	2735 (79.4)		1928 (82.4)	1903 (78.7)		1058 (84.2)	865 (81.6)	
≥High school	91 (1.3)	65 (1.9)		48 (2.1)	35 (1.4)		11 (0.9)	16 (1.5)	
Occupation, No. (%)			0.06			0.50			0.10
Farmer	6328 (92.3)	3141 (91.2)		2170 (92.7)	2253 (93.2)		1186 (94.4)	983 (92.7)	
Others	527 (7.7)	302 (8.8)		171 (7.3)	164 (6.8)		71 (5.6)	77 (7.3)	
BMI in the 1st trimester, kg/m^2, No. (%)			<0.001			<0.001			<0.001
<18.5	680 (9.9)	240 (7.0)		245 (10.5)	163 (6.7)		123 (9.8)	70 (6.6)	
18.5-22.9	4625 (67.5)	2042 (59.3)		1510 (64.5)	1454 (60.2)		832 (66.2)	621 (58.6)	
23.0-27.4	1401 (20.4)	944 (27.4)		520 (22.2)	667 (27.6)		272 (21.6)	299 (28.2)	
≥27.5	149 (2.2)	217 (6.3)		66 (2.8)	133 (5.5)		30 (2.4)	70 (6.6)	
GWG rate in the 2nd/3rd trimester, kg/week, mean (SD)	0.43 (0.17)	0.46 (0.18)	<0.001	0.44 (0.18)	0.45 (0.18)	0.06	0.42 (0.17)	0.44 (0.19)	0.02
Missing, No. (%)	225 (3.3)	81 (2.4)		84 (3.6)	49 (2.0)		55 (4.4)	27 (2.5)	
Anemia in mid-pregnancy, No. (%)			0.90			<0.001			0.60
Yes	410 (6.0)	204 (5.9)		203 (8.7)	128 (5.3)		70 (5.6)	66 (6.2)	
No	6410 (93.5)	3221 (93.6)		2122 (90.6)	2279 (94.3)		1181 (94.0)	989 (93.3)	
Missing	35 (0.5)	18 (0.5)		16 (0.7)	10 (0.4)		6 (0.5)	5 (0.5)	

Table S2 Maternal and offspring characteristics by mode of delivery (for outcomes other than obesity, continued)

Characteristics	Pneumonia			Anemia			CBCL		
	**SVD (n=6855)**	**CDMR (n=3443)**	***P* value**	**SVD (n=2341)**	**CDMR (n=2417)**	***P* value**	**SVD (n=1257)**	**CDMR (n=1060)**	***P* value**
Supplementation during pregnancy, No. (%)			0.80			0.90			0.07
Folic acid	2258 (32.9)	1157 (33.6)		774 (33.1)	814 (33.7)		394 (31.3)	368 (34.7)	
Iron-folic acid	2330 (34.0)	1153 (33.5)		794 (33.9)	810 (33.5)		425 (33.8)	368 (34.7)	
Multiple micronutrients	2267 (33.1)	1133 (32.9)		773 (33.0)	793 (32.8)		438 (34.8)	324 (30.6)	
Level of delivery hospital, No. (%)			<0.001			<0.001			0.005
Provincial/city	541 (7.9)	206 (6.0)		114 (4.9)	131 (5.4)		112 (8.9)	63 (5.9)	
County/district	5259 (76.7)	2528 (73.4)		1800 (76.9)	1572 (65.0)		937 (74.5)	783 (73.9)	
Township/village	1055 (15.4)	708 (20.6)		427 (18.2)	713 (29.5)		208 (16.5)	213 (20.1)	
Missing	0 (0)	1 (0.03)		0 (0)	1 (0.04)		0 (0)	1 (0.1)	
Medical insurance, No. (%)			0.01			0.70			0.30
Yes	6076 (88.6)	3104 (90.2)		2174 (92.9)	2251 (93.1)		1104 (87.8)	945 (89.2)	
No	769 (11.2)	330 (9.6)		164 (7.0)	161 (6.7)		120 (9.5)	87 (8.2)	
Missing	10 (0.1)	9 (0.3)		3 (0.1)	5 (0.2)		33 (2.6)	28 (2.6)	
**Offspring Details**									
Male	3543 (51.7)	1783 (51.8)	0.90	1158 (49.5)	1261 (52.2)	0.07	623 (49.6)	555 (52.4)	0.20
Birth weight, g, No. (%)			0.002			<0.001			0.05
<2500	82 (1.2)	19 (0.6)		44 (1.9)	10 (0.4)		19 (1.5)	6 (0.6)	
2500-3999	6773 (98.8)	3424 (99.4)		2297 (98.1)	2407 (99.6)		1238 (98.5)	1054 (99.4)	
Feeding pattern before 6 months, No. (%)			0.80			0.003			0.70
Exclusive feeding	5657 (82.5)	2858 (83.0)		1904 (81.3)	2035 (84.2)		1021 (81.2)	870 (82.1)	
Mixed feeding	971 (14.2)	479 (13.9)		381 (16.3)	313 (12.9)		154 (12.3)	131 (12.4)	
Formula feeding	218 (3.2)	103 (3.0)		53 (2.3)	67 (2.8)		49 (3.9)	34 (3.2)	
Missing	9 (0.1)	3 (0.1)		3 (0.1)	2 (0.1)		33 (2.6)	25 (2.4)	

Table S2 Maternal and offspring characteristics by mode of delivery (for outcomes other than obesity, continued)

**Characteristics**	**Pneumonia**			**Anemia**			**CBCL**		
	**SVD (n=6855)**	**CDMR (n=3443)**	***P* value**	**SVD (n=2341)**	**CDMR (n=2417)**	***P* value**	**SVD (n=1257)**	**CDMR (n=1060)**	***P* value**
Age at the follow-up visit, month, No. (%) or Mean (SD)			<0.001			<0.001			0.006
18-29	920 (13.4)	693 (20.1)		420 (17.9)	459 (19.0)		588 (46.8)	445 (42.0)	
30-35	1522 (22.2)	864 (25.1)		515 (22.0)	609 (25.2)		214 (17.0)	194 (18.3)	
36-41	1289 (18.8)	690 (20.0)		430 (18.4)	497 (20.6)		117 (9.3)	144 (13.6)	
42-47	1867 (27.2)	738 (21.4)		580 (24.8)	520 (21.5)		198 (15.8)	176 (16.6)	
48-60	1257 (18.3)	458 (13.3)		396 (16.9)	332 (13.7)		140 (11.1)	101 (9.5)	

SVD – spontaneous vaginal delivery, CDMR – cesarean delivery on maternal request, CBCL – Child Behavior Checklist, BMI – body mass index, GWG – gestational weight gain, IQR – interquartile range, SD – standard deviation

Comparisons between groups were made by Student's *t*-test for gestational age and GWG rate, Mann-Whitney U test for maternal age, and chi-square test for other variables.

Table S3 Crude and adjusted odds ratios for multiple child health outcomes by mode of delivery (complete case analysis)

**Outcomes**	**Delivery Mode**	**No. of Events/No. of Children (%)**	**Crude OR (95% CI)**	***P*-value**	**Adjusted OR (95% CI)***	***P*-value**	**Adjusted OR (95% CI)†**	***P*-value**
Obesity	SVD	221/6608 (3.3)	1 [Reference]		1 [Reference]		1 [Reference]	
	CDMR	156/3473 (4.5)	**1.36 (1.10-1.68)**	0.004	**1.42 (1.14-1.76)**	0.002	**1.48 (1.18-1.84)**	<0.001
Pneumonia	SVD	263/6611 (4.0)	1 [Reference]		1 [Reference]		1 [Reference]	
	CDMR	141/3350 (4.2)	1.06 (0.86-1.31)	0.581	1.13 (0.91-1.41)	0.268	1.15 (0.93-1.44)	0.199
Anemia	SVD	113/2237 (5.1)	1 [Reference]		1 [Reference]		1 [Reference]	
	CDMR	181/2353 (7.7)	**1.57 (1.23-2.00)**	<0.001	**1.64 (1.27-2.11)**‡	<0.001	**1.69 (1.31-2.18)**‡	<0.001
Internalizing Problems	SVD	126/1168 (10.8)	1 [Reference]		1 [Reference]		1 [Reference]	
	CDMR	87/1000 (8.7)	0.79 (0.59-1.05)	0.104	0.81 (0.60-1.09)‡	0.169	0.83 (0.61-1.11)‡	0.211
Externalizing Problems	SVD	51/1168 (4.4)	1 [Reference]		1 [Reference]		1 [Reference]	
	CDMR	36/1000 (3.6)	0.82 (0.53-1.26)	0.365	0.90 (0.57-1.41)‡	0.639	0.91 (0.58-1.44)‡	0.697
Total Problems	SVD	91/1168 (7.8)	1 [Reference]		1 [Reference]		1 [Reference]	
	CDMR	70/1000 (7.0)	0.89 (0.64-1.23)	0.484	0.93 (0.67-1.31)‡	0.685	0.93 (0.67-1.31)‡	0.696

SVD – spontaneous vaginal delivery, CDMR – cesarean delivery on maternal request, OR – odds ratio, CI – confidence interval

*Adjusted for maternal age at delivery (year, continuous), education (≤primary, secondary, or ≥high school), occupation (farmer or not), gestational age (week, continuous), body mass index in the 1st trimester (<18.5, 18.5-22.9, 23.0-27.4, or ≥27.5 kg/m^2), gestational weight gain rate in the 2nd/3rd trimester (kg/week, in quintiles), and micronutrient supplementation (folic acid, iron-folic acid, or multiple micronutrients); child's gender (male or female), birth weight (g, continuous), age at the follow-up visit (month, continuous), and feeding pattern before 6 months old (exclusive breastfeeding, mixed feeding, or formula feeding).

†Additionally adjusted for level of delivery hospital (provincial/city, county/district, or township/village level) and medical insurance status (yes or no).

‡Additionally adjusted for maternal anemia in mid-pregnancy (yes or no).

Table S4 Crude and adjusted odds ratios for multiple child health outcomes by mode of delivery (intention-to-treat analysis)

**Outcomes**	**Delivery Mode**	**No. of Events/No. of Children (%)**	**Crude OR (95% CI)**	***P*-value**	**Adjusted OR (95% CI)***	***P*-value**	**Adjusted OR (95% CI)†**	***P*-value**
Obesity	Planned VD	238/6945 (3.4)	1 [Reference]		1 [Reference]		1 [Reference]	
	Planned CDMR	160/3597 (4.4)	**1.31 (1.07-1.61)**	0.009	**1.36 (1.10-1.68)**	0.005	**1.40 (1.13-1.74)**	0.002
Pneumonia	Planned VD	270/6949 (3.9)	1 [Reference]		1 [Reference]		1 [Reference]	
	Planned CDMR	144/3478 (4.1)	1.07 (0.87-1.31)	0.530	1.13 (0.91-1.40)	0.274	1.15 (0.93-1.43)	0.205

VD – vaginal delivery, CDMR – cesarean delivery on maternal request, OR – odds ratio, CI – confidence interval

*Adjusted for maternal age at delivery (year, continuous), education (≤primary, secondary, or ≥high school), occupation (farmer or not), gestational age (week, continuous), body mass index in the 1st trimester (<18.5, 18.5-22.9, 23.0-27.4, or ≥27.5 kg/m^2), gestational weight gain rate in the 2nd/3rd trimester (kg/week, in quintiles), and micronutrient supplementation (folic acid, iron-folic acid, or multiple micronutrients); child's gender (male or female), birth weight (g, continuous), age at the follow-up visit (month, continuous), and feeding pattern before 6 months old (exclusive breastfeeding, mixed feeding, or formula feeding).

†Additionally adjusted for level of delivery hospital (provincial/city, county/district, or township/village level) and medical insurance status (yes or no).

Table S5 Crude and adjusted odds ratios for childhood obesity and anemia by mode of delivery (restricted to women who had normal BMI in the 1st trimester and normal gestational weight gain rate in the 2nd/3rd trimester)

Outcomes	Imputation	Delivery Mode	No. of Events/No. of Children (%)	Crude OR (95% CI)	*P*-value	Adjusted OR (95% CI)*	*P*-value	Adjusted OR (95% CI)†	*P*-value
Obesity	Yes	SVD	110/2943 (3.7)	1 [Reference]		1 [Reference]		1 [Reference]	
		CDMR	63/1255 (5.0)	1.36 (0.99-1.87)	0.06	1.33 (0.94-1.87)	0.11	**1.43 (1.01-2.02)**	0.04
	No	SVD	102/2802 (3.6)	1 [Reference]		1 [Reference]		1 [Reference]	
		CDMR	60/1210 (5.0)	1.38 (1.00-1.91)	0.05	1.34 (0.95-1.89)	0.09	**1.45 (1.02-2.05)**	0.04
Anemia	Yes	SVD	33/940 (3.5)	1 [Reference]		1 [Reference]		1 [Reference]	
		CDMR	38/881 (4.3)	1.24 (0.77-1.99)	0.38	1.25 (0.75-2.08)‡	0.38	1.32 (0.79-2.20)‡	0.30
	No	SVD	32/888 (3.6)	1 [Reference]		1 [Reference]		1 [Reference]	
		CDMR	38/853 (4.5)	1.25 (0.77-2.02)	0.37	1.25 (0.75-2.08)‡	0.39	1.32 (0.79-2.20)‡	0.30

SVD – spontaneous vaginal delivery, CDMR – cesarean delivery on maternal request, OR – odds ratio, CI – confidence interval

*Adjusted for maternal age at delivery (year, continuous), education (≤primary, secondary, or ≥high school), occupation (farmer or not), gestational age (week, continuous), body mass index in the 1st trimester (kg/m^2, continuous), gestational weight gain rate in the 2nd/3rd trimester (kg/week, continuous), and micronutrient supplementation (folic acid, iron-folic acid, or multiple micronutrients); child's gender (male or female), birth weight (g, continuous), age at the follow-up visit (month, continuous), and feeding pattern before 6 months old (exclusive breastfeeding, mixed feeding, or formula feeding).

†Additionally adjusted for level of delivery hospital (provincial/city, county/district, or township/village level) and medical insurance status (yes or no).

‡Additionally adjusted for maternal anemia in mid-pregnancy (yes or no).

Figure S1 Flowchart of inclusion and exclusion in the intention-to-treat analysis.

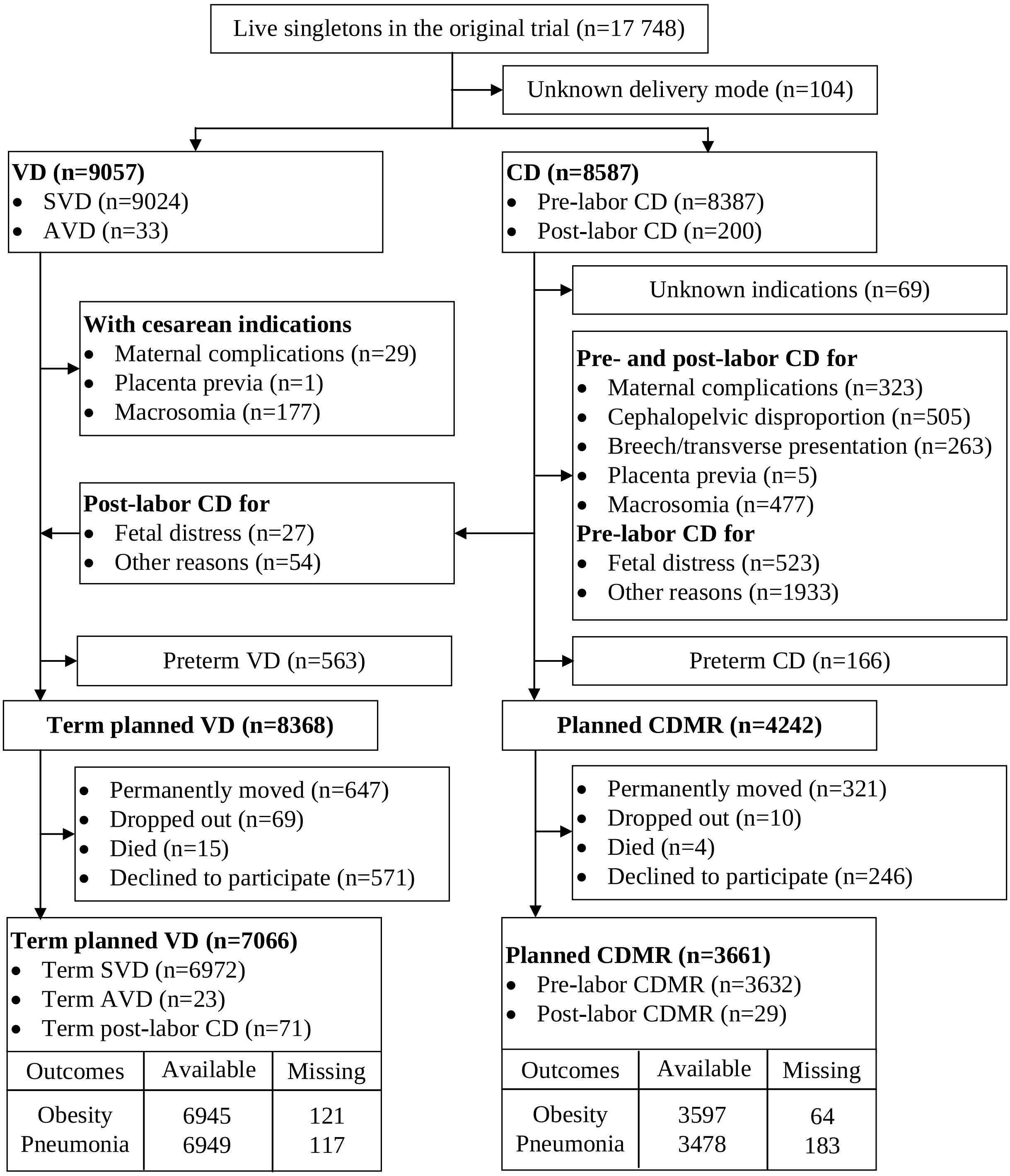

VD – vaginal delivery, SVD – spontaneous vaginal delivery, AVD – assisted vaginal delivery, CD – cesarean delivery, CDMR – cesarean delivery on maternal request

Figure S2 Probability of anemia in different delivery modes by child's age.

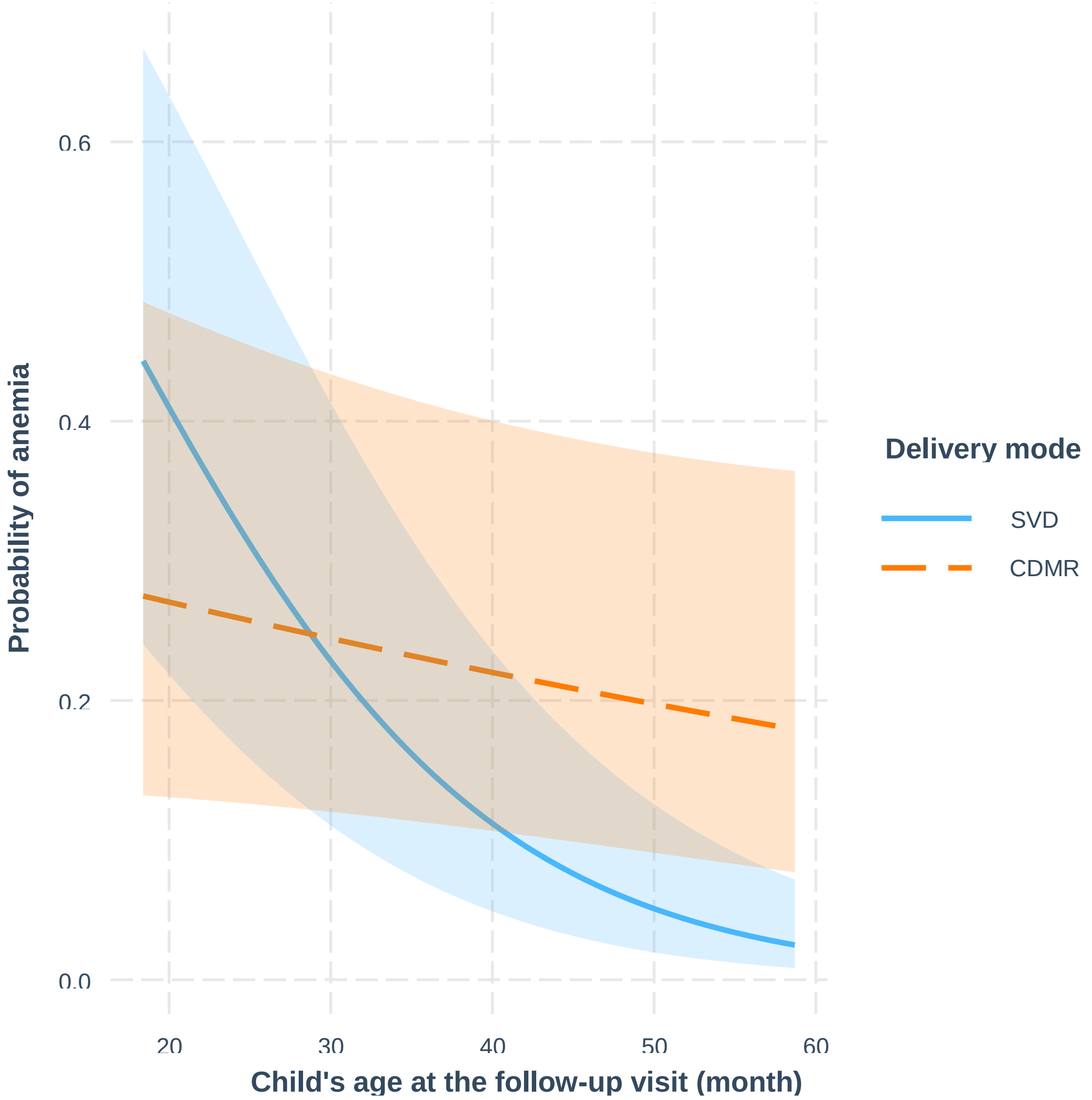

SVD – spontaneous vaginal delivery, CDMR – cesarean delivery on maternal request

The logistic model was adjusted for maternal age at delivery (year, continuous), education (≤primary, secondary, or ≥high school), occupation (farmer or not), gestational age (week, continuous), body mass index in the 1st trimester (<18.5, 18.5-22.9, 23.0-27.4, or ≥27.5 kg/m^2), gestational weight gain rate in the 2nd/3rd trimester (kg/week, in quintiles), maternal anemia in mid-pregnancy (yes or no), micronutrient supplementation (folic acid, iron-folic acid, or multiple micronutrients), level of delivery hospital (provincial/city, county/district, or township/village level), and medical insurance status (yes or no); child's gender (male or female), birth weight (g, continuous), age at the follow-up visit (month, continuous), and feeding pattern before 6 months old (exclusive breastfeeding, mixed feeding, or formula feeding).
